# The Studies in Constructing Yeast Cell Factories for the Production of Fatty Acid Alkyl Esters

**DOI:** 10.3389/fbioe.2021.799032

**Published:** 2022-01-11

**Authors:** Yang Zhang, Xiao Guo, Huaiyi Yang, Shuobo Shi

**Affiliations:** ^1^ Beijing Advanced Innovation Center for Soft Matter Science and Engineering, College of Life Science and Technology, Beijing University of Chemical Technology, Beijing, China; ^2^ CAS Key Laboratory of Microbial Physiological and Metabolic Engineering, State Key Laboratory of Microbial Resources, Institute of Microbiology, Chinese Academy of Sciences, Beijing, China

**Keywords:** yeast, metabolic engineering, esters, enzyme engineering, pathway engineering

## Abstract

Fatty acid alkyl esters have broad applications in biofuels, lubricant formulas, paints, coatings, and cosmetics. Traditionally, these esters are mostly produced through unsustainable and energy-intensive processes. In contrast, microbial production of esters from renewable and sustainable feedstocks may provide a promising alternative and has attracted widespread attention in recent years. At present, yeasts are used as ideal hosts for producing such esters, due to their availability for high-density fermentation, resistance to phage infection, and tolerance against toxic inhibitors. Here, we summarize recent development on the biosynthesis of alkyl esters, including fatty acid ethyl esters (FAEEs), fatty acid short-branched chain alkyl esters (FASBEs), and wax esters (WEs) by various yeast cell factories. We focus mainly on the enzyme engineering strategies of critical wax ester synthases, and the pathway engineering strategies employed for the biosynthesis of various ester products. The bottlenecks that limit productivity and their potential solutions are also discussed in this review.

## Introduction

Fatty acid alkyl esters are produced from (fatty) alcohols and (fatty) acids via esterification (Cruz et al., 2020). These esters are structurally diverse according to the chain length of the fatty acid moiety and the alcohol moiety (Menendez-Bravo et al., 2017). Some fatty acid alkyl esters, such as FAEEs and FASBEs, consist of a long-chain acyl moiety with a short-chain alkyl moiety, and these esters are suitable for use as biofuel molecules. FAEE is considered as one of the main components of biodiesel, which provides an environmentally attractive alternative to fossil diesel (Hill et al., 2006; Kuan et al., 2019); FASBE possesses a methyl branch in the alcohol portion, which affords a lower cloud point than FAEE, thus enabling superior use for energy applications in cold-climates (Wang et al., 2012). Traditionally, bio-esters with the short-chain alkyl moieties mentioned above are industrially produced from plants, animals, or waste cooking oils by transesterification with alcohols (Leung et al., 2010) in the presence of a base, an acid, or an enzyme catalyst. However, the high cost and low availability of edible oils limit the large-scale application of bio-esters, and more sustainable ways need to be explored to address this problem (Jetter and Kunst, 2008; Santori et al., 2012; Chen et al., 2018).

Wax esters (WEs) are long-chain fatty acid alkyl esters composed of a long-chain acyl moiety and a long-chain alkyl moiety, commonly used in the cosmetic, lubricant, and food industries. For example, a jojoba-like WE is a high-value ester of a long-chain fatty acid (C20-C24) and fatty alcohol (C20-C24), and is widely used in cosmetic products [e.g., moisturizers, shampoos, and conditioners] (Kalscheuer et al., 2006; Grand View Research, 2020). Commercial WEs from different sources have different applications. In general, WEs with longer chains are more valuable, since quantities are limited, and in the main are extracted from a few species of plants or animals [e.g., desert shrub jojoba and sperm whale] (Soong et al., 2021).

For the past few decades, microbial production routes have offered new opportunities for producing various fatty acid alkyl esters with different chain length distribution and considerable progress has been made (Steen et al., 2010; Soong et al., 2021). Within these cell factories, yeast cells are promising alternative hosts for the production of such esters due to their robust growth under harsh fermentation conditions such as low pH levels, and a high tolerance against phage contamination and various toxic inhibitors (Krivoruchko et al., 2011). On the other hand, a plentiful of researches in reprogramming yeast metabolism have been reported for production of many kinds of fatty acid derived compounds (Gajewski et al., 2017; Li et al., 2020; Wu et al., 2021; Yu et al., 2018). In this review, we summarize recent development on the biosynthesis of FAEEs, FASBEs, and WEs by yeasts, and focus on the efforts that researchers have made on pathway design, pathway optimization, and enzyme engineering for improving the productivity of these esters.

## Pathway Design and Optimization

**FIGURE 1:**
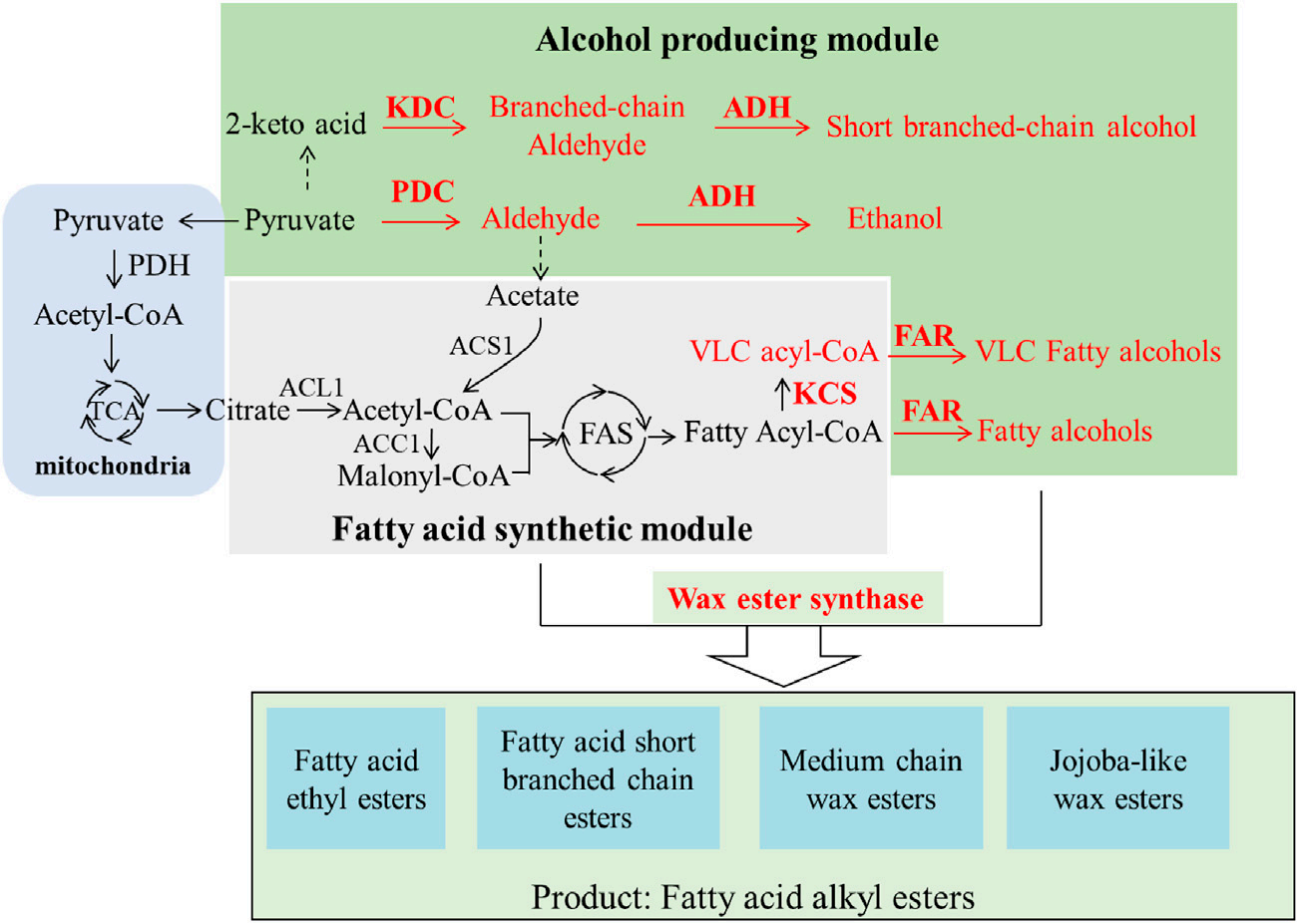
Schematic of the synthetic pathway of various bio-esters. KDC, α-ketoacid decarboxylase; ADH, ethanol dehydrogenase; FAR, fatty acyl-CoA reductase; PDC, pyruvate decarboxylase; KCS, fatty acid elongase.

Generally, the synthetic pathways of fatty acid alkyl esters include an alcohol-producing module and a fatty acid synthetic module, which can be esterized by wax ester synthase (WS) (Figure 1). A great deal of effort has been made to construct such pathways for producing tailored esters. Various metabolic engineering strategies such as eliminating competing pathways and overexpressing the key enzymes involved in the biosynthetic pathways have been utilized to boost the production of such esters.

### Constructing Yeast Cell Factories for the Production of FAEEs

In *S. cerevisiae*, ethanol can be produced through an endogenous pathway that includes a pyruvate decarboxylase (PDC) and an alcohol dehydrogenase (ADH). Taking advantage of this property, several research groups initialized the microbial production of FAEE by introducing only a WS to *S. cerevisiae*. Shi et al. (2012) heterogenously expressed five WSs derived from different organisms and achieved 8.2 mg/L FAEEs using the endogenous ethanol and fatty acid substrates, providing a proof-of-concept in FAEE production through yeast-based fermentation. Later, the same group applied a chromosome engineering method based on delta integration to produce FAEE, and further overexpressed genes encoding an endogenous acyl-CoA binding protein (ACB1) and an NADP^+^-dependent glyceraldehyde-3-phosphate dehydrogenase (gapN) to increase the production. The strategies led to a final titer of more than 40 mg/L (Shi et al., 2014). In addition, Thompson et al. combined metabolic engineering strategies for increasing cytosolic acyl-CoA pools with modification of culture conditions to push lipid production, which led to an FAEE titer of 25 mg/L (Thompson and Trinh, 2014). In doing so, the authors found several limiting factors in fatty acid synthesis, and also pointed out that side reactions of the AtfA acyltransferase should be eliminated (Thompson and Trinh, 2014). In parallel, Lian et al. constructed a reversal β-oxidation cycle in *S. cerevisiae*, which led to the synthesis of medium-chain FAEEs (MCFAEEs) (Lian and Zhao, 2014). This work showed the flexibility to produce FAEEs with different carbon chain lengths, demonstrating a broad application platform for the synthesis of fatty acid alkyl esters in *S. cerevisiae.* On the other hand, Yu et al. (2012) minimized the glycerol synthesis pathway and produced 520 mg/L FAEEs from glycerol with additional exogenous fatty acids. This is to date the highest titer in *S. cerevisiae*, but exogenous fatty acids were required in the research.

The above work on FAEE production indicated that an insufficient supply of fatty acid precursors in *S. cerevisiae* may limit FAEE production. For this reason, oleaginous yeasts were considered better hosts for FAEE production due to their ability to produce abundant fatty acid precursors, e.g., *Yarrowia lipolytica* and *Rhodosporidium toruloides*. Xu et al. (2016) introduced and targeted WS to the endoplasmic reticulum of the well-studied *Y. lipolytica*, and achieved 142.5 mg/L FAEEs. Interestingly, this work also found that targeting enzymes to different cellular compartments gave distinct FAEE product profiles, e.g., ER targeting WS led to longer chain-length. Later, Gao et al. (2018) developed an engineered strain with a fine-tuned expression level of WS, and obtained 1.18 g/L FAEEs when adding exogenous ethanol. Recently, Zhang et al. (2021) engineered *R. toruloides* for FAEE production, and the maximum titer reached 10 g/L with additional exogenous ethanol. The study showed a high capacity for FAEEs production of *R. toruloides,* implicating its abundant metabolic ability to produce fatty acid derivates. To avoid supplying exogenous ethanol, Yu et al. constructed an ethanol-producing pathway in *Y. lipolytica* by introducing a PDC and an ADH from *S. cerevisiae*. However, the FAEE titer was less than 1 mg/L. When 2% ethanol was added to the culture medium, the titer of FAEE reached 360 mg/L (Yu et al., 2020). The result indicates ethanol insufficiency is a major bottleneck in the development of *Y. lipolytica* as an efficient FAEE producer.

### Constructing Yeast Cell Factories for the Production of FASBEs

Although FASBEs have better properties than biodiesels, there is little research on the biosynthesis of FASBEs. Teo et al. firstly reported the production of FASBEs in *S. cerevisiae*, and overexpressed *Ilv2*, *Ilv5,* and *Ilv3*, the key genes in the 2-keto acid synthetic pathway, to increase the supply of branched-chain alcohol. A maximum titer of 230 mg/L FASBEs was achieved in the recombinant strain, including ethyl, isobutyl, isoamyl, and active amyl esters (Teo et al., 2015). Meanwhile, Tao et al. (2015) tried to produce FASBEs in *Pichia pastoris* by introducing a similar pathway which led to a titer of 169 mg/L.

Learning from previous attempts at FAEE production, there is great potential to develop oleaginous yeasts to construct FASBE cell factories in the future, although more metabolic engineering strategies are required to improve the supply of alcohols for the production of FASBEs. For example, Avalos et al. (2013) greatly improved the production of branched-chain alcohols through mitochondria localization, which may provide guidance for engineering the alcohol-producing module to enhance subsequent FASBE production.

### Constructing Yeast Cell Factories for the Production of WEs

Wax esters, especially the jojoba-like wax esters (mainly esters with carbon chain length between C40 and C42), are hard to obtain from natural sources (Jetter and Kunst, 2008). There have been several instances of research relating to engineering yeasts to produce wax esters with tailored chain lengths. Wenning et al. (2017) heterologously expressed FAR (fatty acyl-CoA reductase) and WS derived from different organisms and achieved medium-chain WEs*.* In the same report, they also enabled the synthesis of jojoba-like WEs up to a chain length of C42 by introducing a fatty acid elongase, Elo2p, from *S. cerevisiae*. A further study reported by the same group co-expressed a fatty acid elongase from *Crambe abyssinica* (*Ca*KCS) and a yeast-derived fatty acid desaturase (FAD), Ole1p, that led to the production of diunsaturated WEs (up to C46:2-WE) (Wenning et al., 2019).

After the demonstration of WE production in *S. cerevisiae*, similar strategies and techniques were also extended to oleaginous yeasts. Gao et al. engineered *Y. lipolytica* for very long chain WEs (C32-C42) production by introducing various elongases together with WS and FAR. The WE titer was then improved by impairing the efficiency of the β-oxidation pathway and overexpressing genes related to the accumulation of fatty acyl-CoA. Through scaled-up fermentation, the titer of WEs increased to 2.0 g/L (Gao et al., 2020). Soong et al. (2021) used waste cooking oil as the carbon substrate of an engineered *Y. lipolytica* cell factory containing WS and FAR, and enabled the production of medium-chain WEs with a titer of 7.6 g/L. These strategies may enable large-scale biomanufacturing of jojoba-like WEs in the future and potentially lay a solid foundation to produce other WEs containing very-long-chain FAs.

## Enzyme Discovery and Engineering

WS has long been the widely used enzyme for fatty acid alkyl ester production. Thus, we mainly focused on the development of WS or its variants. The discovery of different WSs with diverse specificities for alcohol and acyl-CoA substrates enabled the biosynthesis of tailored bio-esters (Xu et al., 2021; Holtzapple and Schmidt-Dannert, 2007). Further enzyme engineering strategies enhanced the properties of WS and improved the production of target products (Barney et al., 2013; Barney et al., 2015).

### Enzyme Discovery and Characterization

Until now, many WSs have been identified and characterized from various organisms, e.g., AtfA from *A. baylyi* (Kalscheuer and Steinbuchel, 2003)*, Ha*WS from sunflower (Shalini and Martin, 2020), *Eg*WS from *Euglena gracilis* (Teerawanichpan and Qiu, 2010), *Cz*WS1 from *Chromochloris zofingiensis* (Xu et al., 2021), and *Mh*WS2 from *Marinobacter hydrocarbonoclasticus* (Miklaszewska et al., 2018). In brief, the discovery of these WSs were usually performed by the use of sequence alignment and phylogenetic analysis, including BLAST (http://www.ncbi.nlm.nih.gov/BLAST), CLUSTALW (Kohli and Bachhawat, 2003), and PASTA (Collins and Warnow, 2018). In recent years, the advances in biosynthetic knowledge and predictive bioinformatics tools may greatly facilitate the discovery of new WSs, such as omics-based metabolic data mining platforms (Liu et al., 2020; Medema et al., 2021; Schorn et al., 2021) and Predictor (https://github.com/ccdmb/predector) (Jones et al., 2021).

Most of the reported WSs belong to a class of bifunctional enzymes, which exhibit both WS and diacylglycerol (DAG): acyl-coenzyme A (CoA) acyltransferase (DGAT) activities. Table 1 summarizes the properties of the reported WSs, and the sequence phylogenetic analysis was shown in Supplementary Figure S1. The functional characterization of WSs opened the possibility of producing tailored bio-esters. e. g., *Cz*WS1 with 18:1-CoA preference may represent a promising target for producing 18:1-enriched WE, which has favorable properties for lubrication (Xu et al., 2021). The isoprenoid WS (WS1/WS2 from *Marinobacter hydrocarbonoclasticus* DSM 8798) has been identified as a way to produce isoprenoid WEs, which would provide energy storage and serve as a biochemical marker in *Marinobacter* species (Holtzapple and Schmidt-Dannert, 2007).

**TABLE 1:** The overview of the properties of reported WSs.

**Name**	**Source**	**Acyl-CoA preference**	**Alcohol preference**	**DGAT activity**	**References**
*Ha*WS	Sunflower (*Helianthus annuus*)	16:0- and 18:0-CoA	C16 and C18 alcohols	Y	Shalini and Martin, (2020)
Ma1	*Marinobacter aquaeolei*	14:0-CoA	C10 and C11 alcohols	Y	Barney et al. (2012)
	*VT8*				
*Cz*WS1	*C. zofingiensis*	18:1-CoA	C16 and C18 alcohols	Y	Xu et al. (2021)
*Mh*WS2	*M. hydrocarbonoclasticus*	14:0-, 18:1-, 18:0-, 12:0- and 16:0-CoA	C10 to C16 alcohols	N	Miklaszewska et al. (2018)
*Ph*WS1	Solanaceae	Saturated very long chain acyl-CoA (C20 and C22)	Medium chain alcohols (C8-C12)	N	King et al. (2007)
*Sc*WS	*Simmondsia chinensis*	14:0-CoA/18:0-CoA	C20:1 alcohols/C14 alcohol	Y	Miklaszewska and Banas, (2016)
*Eg*WS	*Euglena gracilis*	14:0-CoA	C16 alcohol	N	Teerawanichpan and Qiu, (2010)
AtfA	*A. baylyi*	12:0-, 14:0-, 16:0-, 18:0-, 18:1-, and 20:0-CoA	C12, C14, C16, C16:1, C18:0, C18:1 alcohol	Y	Kalscheuer and Steinbuchel, (2003)
*Egu*WS	*Elaeis guineensis*	Saturated very long chain acyl-CoA	Medium chain alcohol	Y	Yuan et al. (2020)
*Tr*WSD4/*Tr*WSD5	*Thraustochytrium roseum*	C12-CoA/C10-CoA	Medium and long chain alcohol	Y	Zhang et al. (2017)
AWAT1/AWAT2	Human	Saturated acyl-CoA/unsaturated acyl-CoA	C10 alcohol/C16 and C18 alcohol	Y	Turkish et al. (2005)
WSD1	Arabidopsis	16:0-CoA	C26 and C28 alcohols	N	Patwari et al. (2019)
					
Atf_G25_	*Streptomyces sp.*G25	12:0- or 16:0-CoA	C12 to C18 alcohol	Y	Rottig et al. (2016)
Atf1/Atf2	*Rhodococcus opacus*	N.A^a^	N.A^a^	Y	Alvarez et al. (2008)
*Pt*WS	*Phaeodactylum tricornutum*	14:0-, 16:0- and 18:0- CoA	Very long chain alcohols	Y	Cui et al. (2018)
WS2	*M. hydrocarbonoclasticus* DSM 8798	Long-chain acyl-CoA	isoprenoid alcohol	Y	Holtzapple and Schmidt-Dannert, (2007)

a N.A.: not available.

### Enzyme Engineering

WS is a large family of enzymes that are generally considered promiscuous and will catalyze the production of various esters from a broad range of different substrates. Therefore, it is of great significance to enhance its properties through enzyme engineering to produce tailored bio-esters.

Random mutagenesis has become a valuable tool in engineering the properties of enzymes as biocatalysts. Rottig et al. obtained several AtfA mutations (Glu15Lys, Trp67Gly, Ala126Asp, Ser374Pro, or Gly378Ser/Asp) with diminished lipid accumulation through random mutagenesis by a mutator strain *E. coli* XL-1 Red (Rottig and Steinbüchel, 2013). Santín evolved a bifunctional WS/DGAT enzyme, tDGAT from *Thermomonospora curvata* towards improved WS activity but weakened DGAT activity through the error-prone PCR method (Santin et al., 2019).

Now a series of approaches learned from rational protein engineering can be implemented to reprogram WS with the desired functions. For example, there have been studies of rational active site modifications to alter alcohol selectivity in WS by using the crystal structure of the phthiocerol dimycocerosyl transferase (PapA5) from *Mycobacterium tuberculosis* (Barney et al., 2013; Barney et al., 2015). In particular, a substitution at the residue of 360 from alanine to isoleucine in *Ma*WS1, a WS from *Marinobacter aquaeolei* VT8 (also named Ma1), resulted in shifted selectivity toward short-chain alcohols (Barney et al., 2013). Furthermore, a corresponding mutation of Ac1 (Ac1-G355I), also known as AtfA, resulted in a similar shift in the substrate profile. The same research group also engineered Ma1 at residues 356 and 405, and found that substitutions of L356A, L356F, L356V, and M405W showed increased selectivity to ethanol, and substitution of M405F showed increased selectivity to isoamyl alcohol. The evolved enzymes may provide the potential to improve the yield of FAEEs or branched alcohol-based esters (Barney et al., 2015).

Later, Zhang et al. (2021) heterogeneously expressed AtfA-G355I in *R. toruloides*, and confirmed that the engineered enzyme increased FAEE production by 20% compared with the wild type AtfA. In parallel, based on two predicted transmembrane domains, Kawelke et al. identified and demonstrated an AWAT2 N36R variant of mouse AWAT2, which led to higher efficiency toward very long-chain acyl-CoA than the wild type (Kawelke and Feussner, 2015). Recent advances in computational techniques have greatly facilitated the process of enzyme engineering that can be learned for future WS engineering. For example, the quantum mechanics-cluster approach is a popular technique for elucidating the enzymatic reaction mechanisms (Himo, 2017); Caver Web is a commonly used tool for identification of access pathway and analysis of ligand transport (Stourac et al., 2019); SoluProt (Hon et al., 2021) and DeepSol (Khurana et al., 2018) are widely used tools for predicting protein solubility. Virtual screening is an ideal tool to assess which proteins amongst a library of variants can better accommodate and catalyze a given substrate of interest (Zhang et al., 2019).

Although there are some successful rational modifications of WS, efficiency was still hindered by limited structural information. In 2018, Petronikolou et al. reported the first structure of the WS/DGAT superfamily the *Ma*WS1 from *M. aquaeolei* VT8 (Petronikolou and Nair, 2018). Guided by the crystal structure, a mutant *Ma*WS1-A144V was generated with ∼3 times more efficiency toward a shorter acyl-CoA (C6-CoA). This work provided guidance for further engineering studies.

## Challenges and Opportunities

So far, microbial production of fatty acid alkyl esters has been considered a promising alternative and sustainable source that is widely used in industry in solvents, plasticizers, biodiesel, coatings, and fuel additives. On the other hand, the metabolic engineering strategies may direct the production of the ester with tailored chain length, which might otherwise require complex processes through chemical methods. Nevertheless, the production of bio-esters through microbial fermentation still faces the following challenges.

1) The unbalanced carbon flux between alcohol and fatty acyl-CoA is one of the main problems in bio-ester production. For example, in *S. cerevisiae*, the FAEE production was limited by the supply of fatty acid precursors (Shi et al., 2012). However, when adding exogenous fatty acid, the production of FAEE was greatly improved (Yu et al., 2012). To date, efforts have been made to enhance the equilibrium of carbon flux between alcohol and fatty acyl-CoA substrates. However, most of the metabolic engineering strategies were based on static control (Shi et al., 2012; Thompson and Trinh, 2014; Valle-Rodriguez et al., 2014; Eriksen et al., 2015; de Jong et al., 2015). Further improvement in production calls for more efficient approaches that could intelligently control the alcohol or fatty acid metabolism. A prominent study constructed FAEE cell factory by employing an acyl-CoA dynamic sensor-regulator system (DSRS) in *E. coli.* The introduced feedback reduced toxic ethanol accumulation and enhanced the stability of the recombinant strain, thereby improving the FAEE production by 3-fold (Zhang et al., 2012). Later, Dabirian et al. (2019) established a fatty acyl-CoA sensor based on FadR in *S. cerevisiae*, identified novel targets enhancing acyl-CoA levels. These sensor-based studies provided perspectives in controlling substrate pools for further constructing ester cell factories. In addition, the recent development on constructing synthetic consortia systems by distributing complex pathways in different cells may provide an efficient strategy to solve the problem of an unbalanced carbon flow. Yu et al. (2020) constructed a synthetic consortium by an ethanol-producing yeast *S. cerevisiae* and an oleaginous yeast *Y. lipolytica*, and this synthetic consortium produced 4.8 mg/L FAEE. The coculture system is expected to be further optimized to result in a significant improvement in FAEE production.
2) The low specificity of WS is another challenge to the production of tailored bio-esters, especially fatty acid short-chain esters. As shown in Table 1, the reported WSs have a low affinity towards short-chain alcohols. Gao et al. (2018), Zhang et al. (2021) reported ethanol concentration as a key factor for FAEE production, and the optimum concentration of exogenous ethanol for FAEE production was 5% (∼40 g/L). So far, several enzyme engineering strategies have been employed to increase selectivity towards short-chain alcohols; however, the effect was not significant (Rottig et al., 2015). Therefore, extensive research on the rational design and engineering of WS is urgently required to further improve activity, stability, and specificity. The fast-growing artificial intelligence algorithms such as Alphafold may provide higher predictability for enzyme engineering (Jumper et al., 2021). In addition, more WSs with different properties are expected to be discovered for tailored bio-esters production.
3) The lack of a high throughput screening method limited enzyme discovery and engineering, and made cell factory construction more tedious. Lobs et al. developed a method for high throughput analysis of microbial short-chain volatile ester biosynthesis through a hydroxylamine/ferric iron reaction (Lobs et al., 2016). The assay was depended on the reaction of esters with hydroxylamine, which produced hydroxamic acid. When ferric iron was added, it reacted with hydroxamic acid, and an iron complex with strong absorbance between 500 and 550 nm was detected. Using the same method, Lee et al. developed a high throughput screening approach for alcohol acyltransferases (AATs) identification. This platform could investigate the alcohol substrate specificity of AATs and recognize the beneficial mutations in the engineered AATs for enhanced ester synthesis (Lee et al., 2021). Lin et al. developed a novel assay method for AATase activity. The method relied on the conversion of acyl-CoA to CoA-SH by AATase, and a subsequent reaction produced CoA-SH to succinyl-CoA by α-KGDH. The coupled α-KGDH reaction reduced NAD^+^ to NADH, thus enabling the spectrophotometric measurement of AATase activity (Lin et al., 2016). Santin et al. (2019) demonstrated a Nile red-based high throughput screening method, which provided an evolution platform for WS/DGAT-like enzymes. Guo et al. (2016) demonstrated a high throughput single cell screening method for lipid production with fluorescence-assisted optofluidic time-stretch microscopy. These studies may provide guidance for the high throughput screening of WSs and ester cell factories. Meanwhile, the identification of the mutations from the high throughput screening will clarify new mechanisms that contribute to the engineering of enzymes.
4) Due to the complexity of metabolic and regulatory networks of the microbial chassis, currently it is difficult to obtain robust phenotypes through rational design and gene perturbation strategies described above. In contrast, the development of adaptive laboratory evolution (ALE) has greatly accelerated the efficiency of chassis engineering, and has been widely used in improving the production of various targeted products. Blount et al. reported an improved *S. cerevisiae* strain with genetic backgrounds favorable to diverse heterologous pathways, such as those for violacein and penicillin biosynthesis through the *in vivo* SCRaMbLE system (Blount et al., 2018). This study demonstrated the *in vivo* rearrangement approach can be used as a valuable approach for strain evolution. Yu et al. (2018) evolved genetic mutations toward fatty acid biosynthesis through ALE under high selection pressure, which fine-tuned the carbon flux and restored cell growth. Li et al. (2021) reprogrammed *Escherichia coli* metabolism using ALE to establish a strain that can efficiently utilize waste cooking oil (WCO) as the sole carbon source to produce medium-chain α,ω-dicarboxylic acids (MCDCAs), namely, monomers of bioplastics. Recently, several noted technologies to target nucleotide diversification have been reported to accelerate the ALE. e. g., Bao et al. (2018) developed a CRISPR-Cas9-and homology-directed-repair-assisted genome-scale engineering method named CHAnGE, which can produce a genome-wide set of yeast mutants with single-nucleotide precision. With the development of synthetic biology and the combination of continuous evolution technology with artificial intelligence technology, the metabolic engineering process will be greatly facilitated, and the production of fatty acid-derived biochemicals will be significantly improved.

In general, fatty acid alkyl esters have broad applications in industries including biofuels, cosmetics, lubricants, etc. Production of such esters through microbial fermentation provides a sustainable route and has achieved much progress in various yeast cells. Nevertheless, there are still many problems to be solved. Understanding the mechanistic detail of carbon flux distribution in microbial chassis and the catalytic mechanism of WS is of great significance for the efficient microbial production of such esters. In recent years, the rapid development of synthetic biology and structural biology will provide new perspectives and revolutions in how we build and engineer new biological systems for human purposes, such as the production of fatty acid alkyl esters.
