# Supplementary material for: The Studies in Constructing Yeast Cell Factories for the Production of Fatty Acid Alkyl Esters
Source: Front Bioeng Biotechnol. 2022 Jan 11;9:799032. doi: 10.3389/fbioe.2021.799032 (PMC8787340; doi:10.3389/fbioe.2021.799032)
Supplement: Supplementary file 1 [file Image1.pdf]

Supplementary Information

The studies in constructing yeast cell factories for the production of fatty acid alkyl esters

Yang Zhang[1, 2], Xiao Guo[1], Huaiyi Yang[2*], Shuobo Shi[1*]

1, Beijing Advanced Innovation Center for Soft Matter Science and Engineering, College of Life Science and Technology, Beijing University of Chemical Technology, Beijing 100029, China
2, CAS Key Laboratory of Microbial Physiological and Metabolic Engineering, State Key Laboratory of Microbial Resources, Institute of Microbiology, Chinese Academy of Sciences, Beijing 100101, China

*** Correspondence:**
Shuobo Shi
shishuobo@mail.buct.edu.cn
Huaiyi Yang
yanghy@im.ac.cn

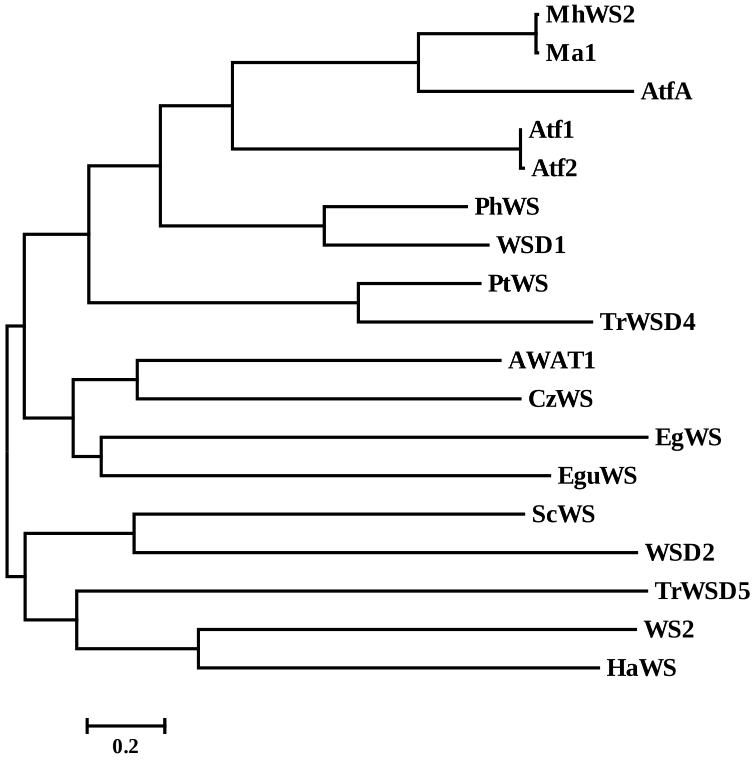

Fig. S1 The sequence phylogenetic analysis of WSs listed in Table 1.
